# Genetic control of sweetness and acidity in blackberry

**DOI:** 10.3389/fpls.2025.1569492

**Published:** 2025-07-25

**Authors:** Carly Godwin, T. Mason Chizk, Carmen Johns, Lacy Nelson, Renee Threlfall, John R. Clark, Margaret L. Worthington

**Affiliations:** ^1^ Department of Horticulture, University of Arkansas, Fayetteville, AR, United States; ^2^ Food Science Department, University of Arkansas, Fayetteville, AR, United States

**Keywords:** *Rubus* subgenus *Rubus*, Rosaceae, GWAS, fruit quality, titratable acidity, soluble solids content, pH, heritability

## Abstract

**Introduction:**

The global blackberry (*Rubus* subgenus *Rubus*) industry has experienced rapid growth during the past 20 years. Even so, many industry stakeholders report complaints from consumers and grocers stating that blackberries are often too tart or not sweet enough for their palate. Studies have shown that most consumers prefer sweet blackberries with relatively low acidity, but little research has been conducted to understand the genetic control of these traits in *Rubus*.

**Methods:**

The objective of this study was to identify quantitative trait loci (QTL) related to sweetness and acidity in autotetraploid blackberry using a genome-wide association study (GWAS). A panel consisting of 301 commercially-available cultivars and University of Arkansas System Division of Agriculture (UA) breeding selections was phenotyped for soluble solids content (SSC), pH, and titratable acidity (TA) from 2019–2021 and genotyped with 65,995 SNPs concentrated in genic regions of the *R. argutus* reference genome.

**Results:**

The average SSC, pH, and TA for the blackberry genotypes evaluated in this study were 10.8%, 3.61, and 0.83%, respectively. Soluble solids content had the lowest broad sense entry mean heritability at 61%, while pH and TA had heritabilities of 67% and 70%, respectively. Four SNPs on chromosome Ra02 were significantly associated with SSC, explaining 7.2% of the variance for this trait. An overlapping QTL for pH and TA, comprised of 187 SNPs significantly associated with one or both traits across a 3.7 Mb region, was discovered on chromosome Ra05. Two peak SNPs located at 4,448,123 and 4,448,155 bp on Ra05 explained 13.6% and 11.5% of observed phenotypic variance for pH and TA, respectively. A gene coding for a sucrose binding protein on chromosome Ra02 was identified as a possible candidate gene for SSC. Seven potential candidate genes for fruit acidity were proposed on chromosome Ra05, including homologs of MYB1, PEPC, malate synthase, and ALMT9.

**Discussion:**

This work provides important insights on the genetic control of sweetness and acidity in fresh-market blackberries. The genotypic and phenotypic datasets reported in this study can be used to develop diagnostic molecular markers and train genomic selection models to expedite selection of new blackberry cultivars with enhanced sweetness and reduced acidity.

## Introduction

As our world becomes more health-conscious, consumers are changing their diet to meet a higher nutritional standard. Due to this shift in health-conscious eating, blackberries are being consumed at an unprecedented rate (Clark and Finn, 2014). The popularity of fresh-market blackberries in the United States is steadily increasing, with imports of $70.67 million in 2023, an increase of 26.4% compared to 2021 imports (United States Department of Agriculture, Economic Research Service, 2024). Even with the rise in blackberry consumption and sales, industry stakeholders report complaints from both consumers and grocers claiming blackberries are often too tart or not sweet enough for their liking (Worthington et al., 2021). The success of fruit cultivars depends upon their consumer appeal, which makes flavor a very important breeding target (Migicovsky, 2020). Studies have shown that most consumers prefer blackberries with a balance between sweet and sour tastes and a medium SSC to TA ratio of 8.93 over fruit with low or high SSC to TA ratios of 6.25 or 10.92 (Dunteman et al., 2018). Therefore, selecting for consistently sweet flavor and balanced acidity is a major objective of fresh-market blackberry breeding programs.

Blackberry flavor is determined by the chemistry of sugars, acids, and volatile organic compounds (VOCs) found in the fruit. Blackberries contain three principal sugars, glucose, fructose, and sucrose, with the most prominent sugars being glucose and fructose. The levels of these sugars present within a given berry change throughout the ripening process (Fan-Chiang and Wrolstad, 2010; Kafkas et al., 2006). The primary organic acids found in blackberries are citric (Finn and Clark, 2017) and malic acid (Kafkas et al., 2006). Historically, fresh-market blackberry breeding goals included thornlessness, erect growth habit, high yield, and fruit quality (Clark et al., 2007). Combining thornlessness and erect growth habit with good flavor was challenging, as the original sources of thornlessness and erect growth habits used in U.S. fresh-market blackberry breeding programs were very tart (Clark, 2005). Despite these challenges, new cultivars with reduced acidity and improved flavor have been developed through years of persistent crossing and selection.

Until recently, very few genomic and genetic resources have been available for the improvement of *Rubus* species (Foster et al., 2019) and very little research has been conducted on the genetic control of sweetness and acidity in *Rubus*. Fortunately, there is a high degree of genome conservation between taxa across the Rosaceae family, and blackberry researchers can build upon genetic research conducted in other diploid fruit crops to accelerate the development of molecular breeding tools (Zurn et al., 2020). QTL associated with reduced acidity have been discovered in apples and peaches. Malic acid (*Ma*) content in apples is controlled by the *Ma* gene in a 65 kb region of the distal end of linkage group L16 that codes for an aluminum-activated malate transporter 9 (ALMT9) (Bai et al., 2012). The ALMT9 protein facilitates diffusion of malate and citrate through vacuolar cell membranes to regulate pH within the vacuole (Etienne et al., 2013). In both *Malus* and *Vitis*, ALMT9 plays a role in the control of fruit acidity (De Angeli et al., 2013; Etienne et al., 2013; Li et al., 2020). A major QTL for acidity in peaches named the *D* locus was mapped to a 0.4 cM genetic interval corresponding to a 100 kb region at the proximal end of LG 5 (Boudehri et al., 2009). An encoding auxin efflux carrier family protein, *Prupe-*5G004300/*ppa006339m*, and *PpRPH*, a putatative small protein, have both been proposed as candidate genes controlling fruit acidity in the *D* locus in peach (Cao et al., 2016; Wang et al., 2021).

While genetic loci associated with major differences in fruit acidity have been mapped in apple and peach, the QTL associated with sweetness in Rosaceae crops tend to have a smaller effect on phenotypic variation and are often inconsistent across environments (Shaw, 2019). Studies of fruit acidity and sweetness show that acidity is much more heritable than sweetness (Lerceteau-Köhler et al., 2012; Shaw, 2019; Zurn et al., 2020). Zhen et al. (2018) identified 25 sugar transporters called *SWEETs* in the apple genome that likely play a role in determining SSC and sweetness. Two of these *SWEET* sugar transporters, *MdSWEET15a* and *MdSWEET9b*, were located in QTL regions associated with variation in SSC and individual sugar content. In peaches, glucose and fructose QTLs on LG 4 and a sucrose QTL on LG 5 were consistently detected across harvest years in a biparental population (Zeballos et al., 2016). A recent study in blackberry identified loci influencing SSC on six of the seven chromosomes, with a QTL identified on *Rubus occidentalis* chromosome Ro01 accounting for a 1.46% difference in SSC across several small biparental populations (Zurn et al., 2020). A gene in the QTL region on Ro01, *qSSC-Ruh-Ch1.1*, had homology with glycosyl-transferase and sucrose synthase genes that have previously been implicated in the accumulation of sugars and starches in other plants (Zurn et al., 2020).

Identifying genetic loci contributing to variation in sweetness and acidity in blackberry could allow breeders to develop genetic markers that can be used during seedling selection and crossing plan optimization. Loci and candidate genes potentially associated with variation in fruit firmness and prickle-free canes in fresh-market blackberry have recently been discovered using GWAS (Chizk et al., 2023, Johns et al., 2025). The objectives of this study were to evaluate titratable acidity, pH, and soluble solids content in a large panel of commercially available fresh-market blackberry cultivars and UA breeding selections and conduct a GWAS to identify marker-trait associations, QTL, and possible candidate genes associated with sweetness and acidity in blackberry.

## Materials and methods

### Plant material and sample collection

A panel of 301 genotypes consisting of 29 commercially available fresh-market blackberry cultivars and 272 UA breeding selections were chosen for GWAS analysis (**Supplementary Table 1**). Two hundred and thirty-two, 234, and 204 genotypes from this panel were harvested and phenotyped in the summers of 2019, 2020, and 2021, respectively. The 301 genotypes in the panel were grown and maintained in 6 m plots at the UA Fruit Research Station (FRS) located in Clarksville, Arkansas. The FRS site is located at 35°31’5”N and long. 93°24’12”W, in USDA hardiness zone 7b, on Linker fine sandy loam. The research plots received regular maintenance of pruning, tipping, integrated pest management, and irrigation.

Blackberry samples were collected from floricane fruit during the summers of 2019, 2020, and 2021, with two harvest dates obtained per genotype each year. The harvest season began in mid-June and continued through the first week of July each year. One pint-sized clamshell (240 g) was collected for each sample. Ripe, marketable berries with no discolored or damaged drupelets were harvested at the shiny-black stage. On rare occasions, poor fruit quality caused by insect pressure prohibited a second harvest for some genotypes. Harvest was delayed at least 24 h after rain events with 5 mm or more of rain, as precipitation can influence fruit quality.

Each clamshell was stored at 5 °C with 90% relative humidity for seven days. After refrigeration, 10-berry samples for each genotype were bagged and frozen in a -12 °C walk-in freezer in preparation for juicing. Frozen 10-berry samples were thawed and squeezed by hand in cheesecloth over a 50-mL centrifuge tube to extract the juice and then frozen again for flavor attribute analysis.

### Evaluation of soluble solids content, pH, and titratable acidity

Samples collected in 2019 were evaluated for SSC (expressed as %) using a tabletop Reichert ABBE Mark II digital refractometer (Reichert Inc., Buffalo, NY). In 2020 and 2021 soluble solids content was analyzed using a handheld PAL-BX/Acid F5 meter for multiple fruits (ATAGO, Tokyo, Japan). Titratable acidity was measured with a Metrohm 862 Compact Titrosampler (Metrohm AG, Herisau, Switzerland) standardized to buffer solutions with a pH of 2.0, 4.0, 7.0, and 10.0 prior to analysis. In 2019, TA was determined by diluting 6 g of juice in 50 mL deionized, degassed water by titration with 0.1 N of sodium hydroxide (NaOH) to a pH endpoint of 8.2. A similar protocol was followed in 2020, using 1 g instead of 6 g of juice diluted in 50 mL deionized, degassed water. TA was not measured for samples collected in 2021. A citric acid standard was used to calculate TA with a milliequivalent factor of 0.064 following the equation:


$$TA\;\left(\%\right)\;=\;\left(\left(mL\;NaOH\;\times \;milliequivalent\;factor\;\times \;100\right)/grams\;of\;sample\right)$$

In 2019, pH was measured with a pH meter fitted to the Metrohm 862 Compact Titrosampler, while pH measurement was conducted with a PH700 Benchtop pH Meter (APERA instruments, Columbus, OH) in 2020 and 2021.

### Analysis of heritability and trait correlations

Best linear unbiased estimates (BLUEs) of SSC, pH, and TA across all years and replicates were calculated for each genotype using the lme4 package in R (Bates et al., 2015). Genotype was considered a fixed effect while harvest year, harvest week nested within year, and genotype by year interaction were modelled as random effects. BLUEs for each trait were also calculated separately for 2019, 2020, and 2021 harvest years with genotype as a fixed effect harvest week as a random effect. BLUEs were used as input for GWAS, following recommendations to avoid reapplying shrinkage from mixed models in both phenotype estimation and association testing (Holland, 2024).

Broad-sense heritability (H) for each trait was calculated using harmonic means for the number of years (*y*) and harvest weeks (*w*) along with estimates of genotypic variance (σ^2g^), genotype by year variance (σ^2gy^), and residual effects (σ^2^). Heritability was calculated as: H = σ^2g^/[σ^2g^ + (σ^2gy^/*y*) + (σ^2^/*yw*)]. Harmonic means for years and replicates were calculated using the dplyr package in R, and variance components were obtained in lme4. Pearson correlation coefficients between SSC, pH, and TA were calculated using the GGally and ggplot2 packages in R.

### Genotyping and SNP calling

A modified cetyltrimethylammonium bromide (CTAB) protocol was used to extract DNA collected from young leaves of each genotype (Porebski et al., 1997). Quantification was completed using the Invitrogen Qubit 1 Fluorimeter dsDNA-BR assay kit (Thermo Fisher Scientific, Waltham, MA), and samples were standardized to 40 ng/µl. CaptureSeq genotyping was performed at RAPiD Genomics (Gainesville, FL). using 35,054 custom-designed biotinylated 120-mer Capture-Seq probes designed using the ‘Hillquist’ (*R. argutus*) reference genome (Brůna et al., 2023). Paired end sequencing was performed on Illumina HiSeq2000 to achieve an average of 5.14 million 150 bp paired-end reads per sample (Chizk et al., 2023). Raw sequence data was cleaned and trimmed then aligned to the ‘Hillquist’ genome using MOSAIK v2.2.30 (Lee et al., 2014) with default parameters. Variant calling was performed using Freebayes v1.3.1 (Garrison and Marth, 2012) using a standard pipeline implemented by RAPiD genomics. SNPs were filtered in VCFtools (Danecek et al., 2011) to produce a file with biallelic markers with a minor allele frequency of ≥ 0.01 and average read depth between three and 750 per sample. Filtered data was then converted to probabilistic tetraploid SNP calls using the multidog function in UpDog v2.0.3 (Gerard et al., 2018). Markers estimated to have a greater than 5% error rate were excluded from the analysis.

### Population structure and linkage disequilibrium

Population structure was inferred using STRUCTURE v.2.3.4 (Pritchard et al., 2000). STRUCTURE analysis was conducted using diploid genotype calls derived from the original VCF prior to conversion to probabilistic tetraploid dosage calls. The analysis was conducted with a population admixture model across a range of putative subpopulations (K = 2 to 8). Each run consisted of a burnin period of 10,000 and 20,000 Markov chain Monte Carlo replications. The optimal K value was selected based on the ΔK method (Evanno et al., 2005) as implemented in Structure Harvester (Earl and vonHoldt, 2012).

Linkage disequilibrium (LD) decay was estimated internally in the GWASpoly package (Rosyara et al., 2016) in R by calculating the average decay of *r^2^* between SNPs using a maximum number of 10,000 SNP pairs and eight degrees of freedom for spline plotting. Lewontin’s *D’* was estimated using the ldsep R package (Gerard, 2021) by sampling SNP pairs spaced at least 100 kb apart within each chromosome. Heatmaps of chromosomal LD patterns were constructed using the LDheatmap R package (Shin et al., 2006).

### GWAS analysis

Association analysis was performed in GWASpoly v2.13 (Rosyara et al., 2016) using SSC, pH, and TA BLUEs calculated for each genotype and filtered biallelic SNP data generated from Capture-Seq. A mixed model was used, incorporating a kinship matrix constructed with the leave-one-chromosome-out (LOCO) method and a fixed Q matrix (K=6) generated by STRUCTURE (Pritchard et al., 2000) as described by Chizk et al. (2023). Minor allele frequency and maximum genotypic thresholds were set to 0.02 and 0.95, respectively. Additive and simplex dominant (1-dom) gene action models were tested.

Quantile-quantile (QQ) plots were were generated to compare observed and expected p-values (**Supplementary Figures 1**, **2–4**), and deviations from the expected uniform distribution indicated p-value inflation in some cases. To address this, empirical significance thresholds were estimated using GWASpoly’s permutation test with 1,000 iterations, which does not assume a theoretical distribution and is robust to test statistic inflation. Using this approach, LOD thresholds selected for SSC were 5.33 (additive), 4.69 (1-dom-alt), and 5.17 (1-dom-ref); for pH, 5.36, 4.65, and 5.21; and for TA, 5.44, 4.69, and 5.24, respectively. Significance thresholds were also calculated separately for each trait in individual year analyses using the same permutation approach, and results are provided in **Supplementary Table 2**.

### Identification of possible candidate genes

Based on patterns of genome-wide LD decay in the GWAS panel reported by Chizk et al. (2023), regions of each chromosome containing significant markers, including a 1 Mb region flanking either side of the significant markers, were investigated for possible candidate genes. Genes with functional annotations related to acidity or sugars in other plant species were considered potential candidates.

## Results

### Phenotypic results

**Figure 1:**
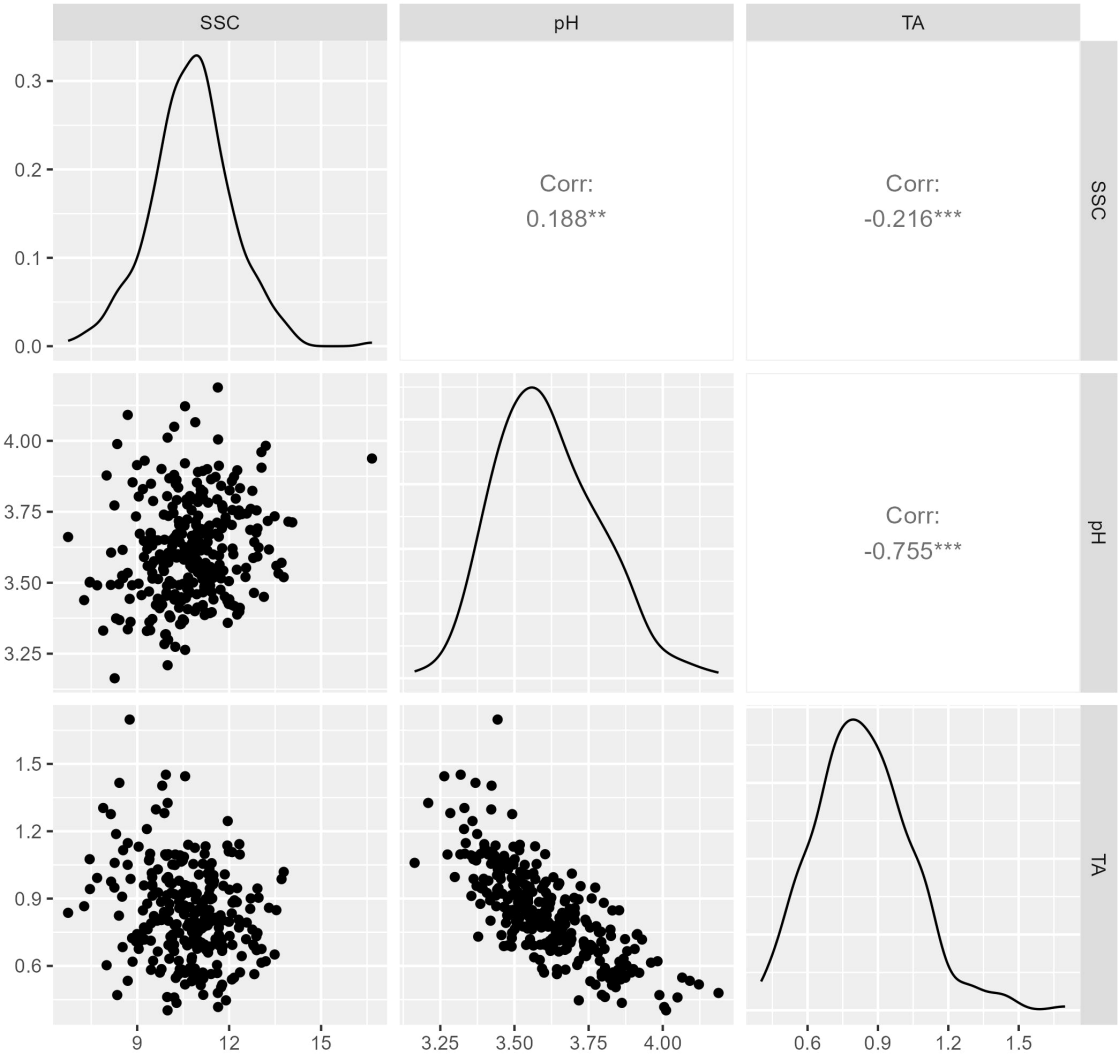
Distribution and correlation of soluble solids content (SSC), pH, and titratable acidity (TA), with TA evaluated in 2019 and 2020 and SSC and pH evaluated in 2019, 2020, and 2021. Diagonal plots show the distributions best linear unbiased estimates (BLUEs) calculated over all years, scatter plots below the diagonal show relationships between traits, and Pearson’s correlation coefficients are above the diagonal.

Data from 1257 replicates collected during 2019, 2020, and 2021 were used to calculate BLUEs for 301 genotypes evaluated for SSC and pH. Best linear unbiased predictions for TA were calculated using 856 replicates from 271 genotypes evaluated in 2019 and 2020 (**Supplementary Table 1**). Overall genotypic BLUEs for SSC and pH were calculated using data obtained in 2019, 2020, and 2021, while BLUEs for TA were calculated with data from the summers of 2019 and 2020. The average normalized BLUEs were 10.8% for SSC, 3.61 for pH, and 0.83% for TA (**Table 1**). The BLUEs for SSC ranged from 6.8% to 16.7%. The range of BLUEs for pH and TA were from 3.16 to 4.18 and 0.40% to 1.69%. BLUEs for all traits were normally distributed (**Figure 1**). Titratable acidity and pH were highly correlated (r = -0.755), while SSC was more weakly correlated with pH (r = 0.188) and TA (r = -0.216) (**Figure 1**). The broad-sense heritabilities of juice attributes were 61% for SSC, 67% for pH, and 70% for TA.

**Table 1:** Minimum, maximum, and mean values for soluble solids content (SSC), pH, and titratable acidity (TA) as normalized best linear unbiased estimates (BLUEs) for fresh-market blackberries grown in Clarksville, AR.

Trait	SSC (%)	pH	TA (% citric)
Maximum	16.7	4.18	1.69
Minimum	6.8	3.16	0.40
Mean	10.8	3.61	0.83

The BLUEs for SSC and pH were calculated using data collected in 2019, 2020, and 2021, and the BLUEs for TA were calculated with data from only 2019 and 2020.

### Genotypic results

The genotypic data used in this study are described in detail in Chizk et al. (2023). Briefly, a total of 81,064 polymorphic markers with an average read depth of 216 were discovered from Capture-Seq genotyping and passed quality filtering thresholds. Of these markers, 75,394 met thresholds for minor allele frequency and maximum genotype frequency and were used in GWAS analyses. Over 99% of SNPs used in the association analysis were in annotated genic regions, and SNP density closely mirrored patterns of gene density across the *R. argutus* genome (Chizk et al., 2023).

### Population structure and linkage disequilibrium

Population structure and LD patterns were characterized using the same genotype dataset described in Chizk et al. (2023). STRUCTURE analysis identified K = 6 as the most likely number of subpopulations based on the ΔK method, with clear differentiation corresponding to known breeding categories in the UA germplasm: primocane-fruiting, floricane-fruiting, and brachytic dwarf types. Although these groups are not reproductively isolated, they align with distinct market niches and breeding objectives. A Q matrix assuming these six admixed subpopulations was used to account for population structure in the GWAS model. Patterns of linkage disequilibrium (LD) revealed genome-wide decay within approximately 5 Mb, with variation among chromosomes and a prominent high-LD block on the distal end of Ra04. For visualization of population structure and LD decay patterns, see Figures 4, 5, and 6 in Chizk et al. (2023).

### Association analysis

**Figure 2:**
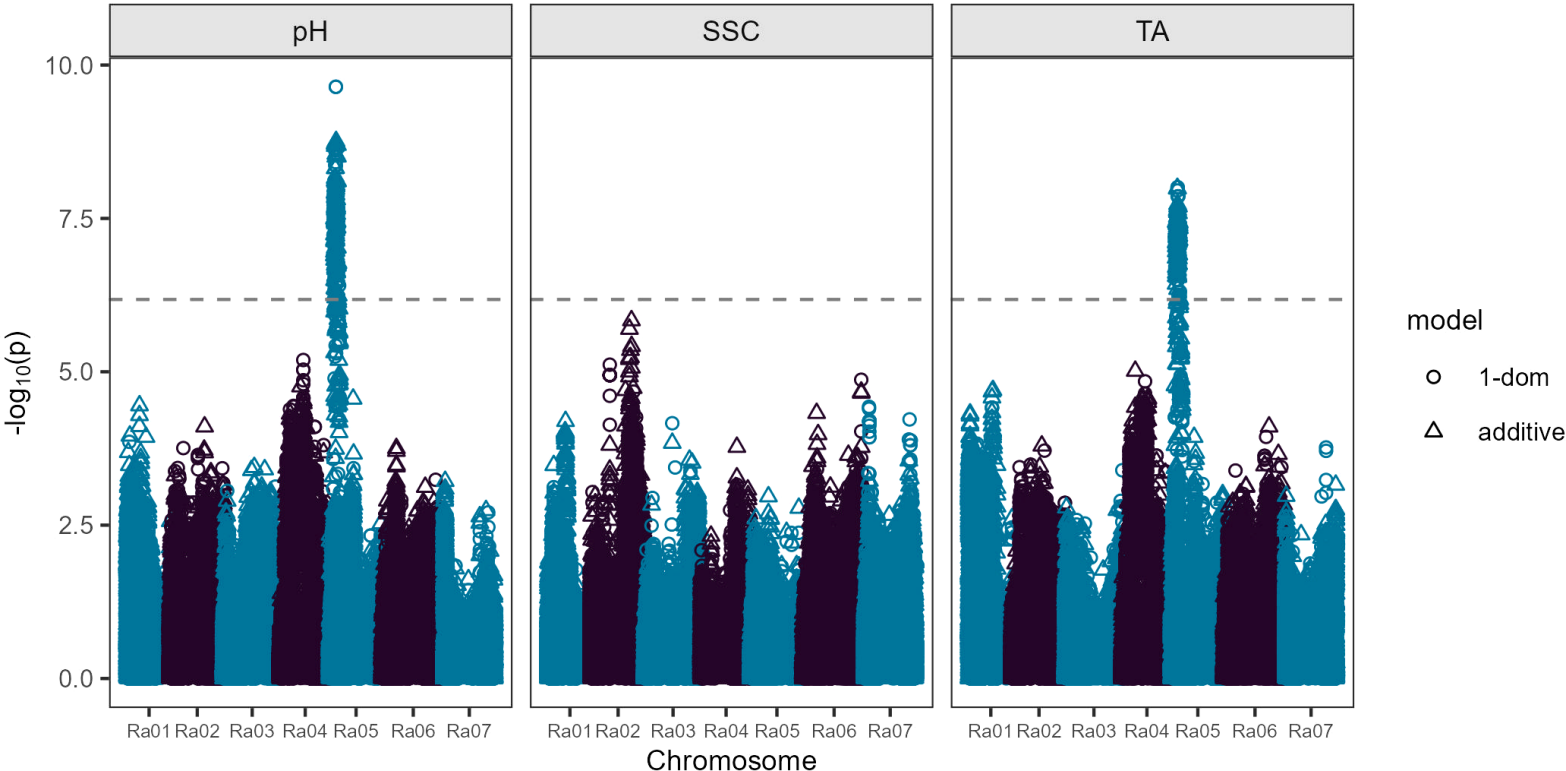
Manhattan plot showing results from a fresh-market blackberry genome-wide association study. Soluble solids content (SSC) and pH phenotypes were calculated using data obtained during 2019, 2020, and 2021, while titratable acidity (TA) phenotypes were calculated using data obtained during 2019 and 2020. The dashed line indicates a Bonferroni-corrected significance threshold. Trait- and model-specific significance thresholds calculated by permutation testing (1,000 iterations) are reported in **Supplementary Table 1**.

Four SNPs located between 27,712,858 and 29,284,319 bp on chromosome Ra02 were significantly associated with SSC under the additive gene action model (**Figure 2**; **Supplementary Table 3**). The most strongly associated SNP at 29,208,288 bp had an LOD score of 5.84 and explained 7.2% of observed phenotypic variance in SSC. In the SSC analysis for individual years, significant marker-trait associations chromosome Ra02 were only detected in one of three years (2021), with 40 significant markers detected between 29,585,041 and 30,601,621 bp. One additional significant SNP was detected on chromosome Ra07 at 4,684,161 bp in 2021. Three significant SNPs were detected on chromosome Ra06 between 38,791,352 and 38,854,419 bp in 2019, and no significant marker-trait associations were detected in 2020 for SSC (**Supplementary Table 4**; **Supplementary Figure 5**).

An overlapping region significantly associated with both TA and pH was found on chromosome Ra05 between 3,384,682 and 7,099,993 bp (**Figure 2**; **Supplementary Table 3**). This approximately 3.7 Mb region contained 187 significant markers under either the additive or simplex dominant models. Of the 187 significant markers, 178 were associated with TA and 176 were associated with pH, with both traits sharing 167 significant markers. One hundred and sixty-three of the 178 markers for TA in this region on Ra05 were significant under both additive and simplex dominance models, while one marker was only significant for the additive model and 14 were significant only under the simplex dominant model. Of the 176 significant markers for pH, one was significant only under the additive model, nine were significant only under the simplex dominant model, and 166 markers were significant under both gene action models.

Two peak markers located at 4,448,123 and 4,448,155 bp on Ra05 had LOD scores of 8.01 for TA and 9.64 for pH in the simplex dominant model. The peak markers each explained 13.6% and 11.5% of observed phenotypic variance for pH and TA, respectively. These peak markers were also significant under the additive model for both traits with LODs of 7.64 for TA and 8.51 for pH for the marker at 4,448,123 bp and LODs of 7.69 for TA and 8.55 for pH for the marker at 4,448,155 bp. The slight differences in LOD scores between these two markers can be attributed to the probabilistic dosage scores used in the GWAS analyses. At both of these peak markers, 252 of the 301 genotypes in the GWAS panel were predicted to be nulliplex, 47 had simplex allele dosage, and two had duplex allele dosages (**Supplementary Table 1**). None of the genotypes in the GWAS panel had triplex or quadruplex dosage for the allele associated with reduced acidity at either peak position. Therefore, it is uncertain if the underlying acidity QTL in this region on Ra05 has additive or dominant gene action.

The significant QTL associated with pH and TA on chromosome Ra05 in the combined analysis across years was detected in two out of three years for pH and in both years for TA in the GWAS analysis for individual years (**Supplementary Table 4**; **Supplementary Figure 5**). In 2019, eight markers on chromosome Ra04 between 6,584,217 and 16,663,919 bp were significantly associated with pH and no significant marker trait associations were detected on chromosome Ra05. There were 36 markers significantly associated with pH on chromosome Ra05 from 3,384,682 to 6,816,272 bp in 2020. In 2021, there were 75 markers significantly associated with pH on chromosome Ra05 from 3,384,682 to 7,009,993 bp and one additional significant marker detected on chromosome Ra06 at 10,346,3339 bp. Two regions significantly associated with TA were detected in 2019, which included 42 markers between 3,969,817 and 7,074,763 bp on chromosome Ra05 and 27 significant markers located between 7,055,714 and 9,827,528 bp on chromosome Ra01. In 2020, the only region significantly associated with TA was on chromosome Ra05, with 38 markers from 3,384,682 to 6,816,974 bp and one additional marker at 16,558,922 bp.

### Possible candidate genes

One possible candidate gene for sweetness and seven possible candidate genes for pH acidity in fresh-market blackberries were identified in this study (**Table 2**). A gene coding for a sucrose binding protein (Ra_g7910) was located 481 kb distally of the significant SNP at 29,284,319 bp on Ra02. Seven possible candidate genes with functional annotations related to fruit acidity in other species were located within 1 Mb of the regions significantly associated with pH and TA between 3,384,682 and 7,099,993 bp on chromosome Ra05. One gene functionally annotated as an ALMT9 proteins (Ra_g20630) was located 337 kb proximally of the QTL on Ra05. Another gene annotated as an ALMT9 (Ra_g21398) was located within the QTL at 7,098,716 bp. The gene Ra_g20727, functionally annotated as a MYB1 transcription factor, was located within the QTL on Ra05 at 3,560,200 bp. A phosphoenolpyruvate carboxylase (PEPC) gene (Ra_g20750) was located at 3,685,932 bp, and three genes coding for malate synthase (Ra_g21026, Ra_g21027, and Ra_g21028) were located at 5,081,414 bp, 5,082,698 bp, and 5,083,558 bp within the TA and pH QTL on Ra05.

**Table 2:** Possible candidate genes for soluble solids content (SSC), titratable acidity (TA), and pH in blackberry in two QTL regions with their location and annotated functions.

Trait	Gene ID	Chromosome	Start bp	Stop bp	Functional annotation	*A. thaliana* homolog
SSC	Ra_g7910	Ra02	29763951	29765632	Sucrose-binding protein	AT3G22640
TA and pH	Ra_g20630	Ra05	3043192	3046890	Vacuolar malate transporter 9 - ALMT9	AT3G18440
TA and pH	Ra_g20727	Ra05	3560200	3560987	Transcription factor protein - MYB1	AT5G49330
TA and pH	Ra_g20750	Ra05	3685932	3686993	Phosphoenolpyruvate carboxylase - PEPC	AT4G10750
TA and pH	Ra_g21026	Ra05	5081414	5082275	Malate synthase	AT5G03860
TA and pH	Ra_g21027	Ra05	5082698	5083511	Malate synthase	AT5G03860
TA and pH	Ra_g21028	Ra05	5083558	5084695	Malate synthase	AT5G03860
TA and pH	Ra_g21398	Ra05	7098716	7102494	Vacuolar malate transporter 9 - ALMT9	AT3G18440

## Discussion

### Insights into phenotypic datasets and heritability

The ranges and means for SSC, TA, and pH obtained in this study were similar to previously published values for fresh-market blackberries. The genotypic BLUEs for SSC reported here ranged from 6.8% to 16.7%. These values were similar to previous research which found that UA blackberry germplasm had SSC values from 4.6% to 16.2% (Zurn et al., 2020) or 9% to 15% (Myers et al., 2023). The mean SSC value reported in this study was 10.8%, above the U.S. minimum standard of 10.0% for blackberries used in the production of beverages containing fruit juice (U.S. Food and Drug Administration, 2020) and comparable to previously reported SSC means for UA fresh-market blackberry populations at 9.9% (Zurn et al., 2020) and mixed fresh-market and processing cultivars at 10.8% (Fan-Chiang and Wrolstad, 2010).

The genotypic BLUEs for pH observed in our fresh-market blackberries ranged from 3.16 to 4.18 with an average of 3.61. A study conducted on processing and fresh-market blackberry cultivars reported a pH range from 2.65 to 3.61 with a mean of 3.19, lower than the pH values observed in our germplasm (Fan-Chiang and Wrolstad, 2010). Other studies conducted with UA blackberry cultivars and selections more closely aligned with the values we observed, with pH values ranging from 3.0 to 3.6 (Threlfall et al., 2016) and 3.0 to 4.2 (Myers et al., 2023). BLUEs for TA in our study ranged from 0.40% to 1.69%, with an average of 0.83%. Fan-Chiang and Wrolstad (2010) reported that TA of processing and fresh-market blackberry germplasm ranged from 0.52% to 2.24% with an average of 1.35%, while studies including only UA fresh-market blackberry germplasm reported TAs ranging from 0.7% to 1.4% (Threlfall et al., 2016) and 0.4% to 1.4% (Myers et al., 2023).

Our study is the first to report broad-sense heritability estimates for SSC, pH, and TA in blackberry, with values of 61%, 67%, and 70%, respectively. Heritabilities for these traits have been reported in related Rosaceae crops. The estimated broad-sense heritability for SSC in peaches was 59% (Rawandoozi et al., 2021), similar to our estimate of 61% in blackberries. However, the broad sense heritability of TA in peaches was 84%, much higher than our estimate for blackberries. Segregation for the major dominant low-acidity allele in the *D* locus likely contributed to the higher heritability estimates for TA in peaches. A major QTL for acidity in peaches named the D locus was mapped to a 0.4 cM genetic interval corresponding to a 100 kb region at the proximal end of LG 5 (Boudehri et al., 2009). Broad-sense heritability estimates for fruit acidity in strawberry were closer to blackberry, with pH and TA estimated at 53% and 65%, respectively (Lerceteau-Köhler et al., 2012).

### Insights on population structure and linkage disequilibrium

A panel of 301 genotypes, comprised of 29 commercially available fresh-market blackberry cultivars and 272 UA breeding selections, was used for GWAS analysis in this study. This panel was genotyped with a set of 65,995 SNPs highly concentrated in genic regions with average read depths exceeding 150x (Chizk et al., 2023). STRUCTURE analysis revealed six subpopulations corresponding to known breeding categories and market niches targeted in the UA breeding program: primocane-fruiting, floricane-fruiting, and brachytic dwarf types.

LD among this population decayed over relatively large distances (Chizk et al., 2023), as is expected for a population of related elite breeding germplasm. LD decay was variable across the genome, with a particularly large LD block located at the end of Ra04 around the prickle-free locus (Chizk et al., 2023; Johns et al., 2025). While our GWAS panel may not represent the full genetic diversity found in broader germplasm collections, its composition offers several distinct advantages for applied breeding. All material is locally adapted to the growing conditions in the Southeastern US, ensuring that identified QTLs are relevant to our target production environment. Most critically, any markers discovered in this panel can be readily integrated into fresh-market breeding pipelines with minimal concern for linkage drag or incompatibility. While slower LD decay in this GWAS panel may reduce mapping resolution, the practical benefits support the utility of this approach.

### Genetic associations with pH and titratable acidity

One hundred and eighty-seven markers in a 3.7 Mb region between 33,846,82 and 7,099,993 bp on chromosome Ra05 were significantly associated with TA and pH. This QTL on Ra05 had a maximum LOD value of 9.64 and explained 13.6% and 11.5% of observed phenotypic variance for pH and TA. In the analysis of individual years’ data pH and TA, significant markers were detected in this region on chromosome Ra05 in two of three years (2020 and 2021) for pH and in both years (2019 and 2020) for TA. The repeated detection of this QTL across years and traits highlights its stability and potential utility as a reliable target for marker-assisted selection aimed at reducing acidity in fresh-market cultivars.

Of the 301 genotypes in the panel, 252 were nulliplex, having four copies of the allele associated with high acidity at the peak LOD positions of 4448123 and 4448155 bp. Forty-seven genotypes were simplex, with one copy of the low acid allele, and only two genotypes were duplex, with two copies each of the low and high acid alleles. Thus, we were unable to determine whether the gene action of the underlying allele was additive or dominant. It is unclear why there were no genotypes with three or more copies of the low acidity allele in the panel, especially considering that reduced acidity is a highly desired trait in fresh-market breeding programs. One possibility that should be further investigated is that there could be a deleterious effect of the low acid allele at higher dosages.

Seven possible candidate genes for the genetic control of blackberry acidity were identified within a 1 Mb region flanking the acidity QTL on Ra05, three for malate synthase, two for ALMT9, and one each for MYB1 and PEPC. The proximal ALMT9 gene, Ra_g20630, was found 341 kb upstream from the QTL, with a second ALMT9 gene, Ra_g21398, located 949 kb distal of the QTL. These ALMT9 genes were considered possible candidate genes for pH and TA in fresh-market blackberries as the *Ma1* candidate gene in the apple acidity locus codes for ALMT9 (Etienne et al., 2013; Liu and Zhou, 2018; Li et al., 2020). Plant ALMTs transport malate across vacuolar cell membranes through facilitated diffusion of an electrical potential gradient (Δ_Ψ_) regulating vacuolar storage and concentration of malate and citrate in cells, with citrate transport occurring far slower than malate (Etienne et al., 2013). Malate transport relies on the presence of a Δ_Ψ_ to occur, with the Δ_Ψ_ expected to decrease as vacuolar pH decreases, effectively closing the transport channels at a low vacuolar pH to prevent over-acidification of already highly acidic vacuoles (Etienne et al., 2013). In apple, the recessive low acid allele, *ma1*, results in a truncated protein with a lost C-terminal domain essential for malate transport activity.

Another possible candidate gene, Ra_g20727, in the acidity QTL region on Ra05 codes for the MYB1 transcription factor. MYB1 regulates the expression of genes that encode vacuolar proton pump subunits, an anthocyanin transporter, and a malate transporter in apples, modulating anthocyanin and malate accumulation in the vacuole (Hu et al., 2016, 2017). A gene, Ra_g20750, encoding PEPC is also located within the QTL region. The PEPC gene was considered a possible candidate for acidity because it is a key enzyme in the pathway responsible for the initial formation of organic acids. Specifically, PEPC catalyzes the reaction causing carboxylation of phosphoenolpyruvate in the cytosol, producing oxaloacetate, which can then be reduced to malate by the cytosolic NAD-dependent malate dehydrogenase (Etienne et al., 2013). Three contiguous genes (Ra_g21026, Ra_g21027, and Ra_g21028) in the QTL region on Ra05 code for malate synthase. Malate synthase is one of five key enzymes involved in the glyoxylate cycle, which facilitates conversion between tri- and dicarboxylates in the glyoxysome (Etienne et al., 2013) and are therefore considered possible candidates for the control of blackberry acidity.

### Genetic associations with soluble solids content

Four SNPs located between 27,712,858 and 29,284,319 bp on chromosome Ra02 were significantly associated with SSC under the additive gene action model (**Figure 2**; **Supplementary Table 3**). The most strongly associated SNP at 29,208,288 bp had an LOD score of 5.84 and explained 7.2% of observed phenotypic variance in SSC. In the SSC analysis for individual years, significant marker-trait associations chromosome Ra02012 were only detected in one of three years (2021), with 40 significant markers detected between 29,585,041 and 30,601,621 bp. One additional significant SNP was detected on chromosome Ra07 at 4,684,161 bp in 2021. Three significant SNPs were detected on chromosome Ra06 between 38,791,352 and 38,854,419 bp in 2019, and no significant marker-trait associations were detected in 2020 for SSC (**Supplementary Table 4**; **Supplementary Figure 5**).

In the combined analysis across years, a single QTL associated with SSC was identified on chromosome Ra02 with four significant SNPs with a peak LOD of 5.84. The most significant SNP at 29,208,288 bp explained 7.2% of observed phenotypic variance in SSC. While the fruit acidity QTL was quite stable across traits and years, significant marker-SSC associations on chromosome Ra02 were only discovered in one of three years (2021) in the analysis of individual years’ data.

Fruit acidity is generally more heritable than sweetness, and QTL for SSC and individual sugars tend to have smaller effects and are often inconsistent across environments (Lerceteau-Köhler et al., 2012; Shaw, 2019; Zurn et al., 2020). Quantitative trait loci associated with variation in SSC have been previously mapped in the *Rubus* (Zurn et al., 2020), *Prunus* (Rawandoozi et al., 2020), *Malus* (Guan et al., 2015), and *Fragaria* (Lerceteau-Köhler et al., 2012) genera of the Rosaceae family. An earlier study conducted with blackberry populations from the UA and United States Department of Agriculture (USDA) Horticultural Crops Production and Genetic Improvement Research Unit breeding programs found 48 significant alleles for SSC mapped to six of the seven base chromosomes of the *Rubus occidentalis* L. genome (Zurn et al., 2020). However, only six of these 48 alleles were significantly associated with sugar content in the UA populations in either year, five on Ro01 and one on Ro04 (Zurn et al., 2020). One QTL, *qSSC-Ruh-ch1.1*, on chromosome Ro01 in the region of a sucrose synthase gene accounted for a 1.46% difference in SSC in the combined UA and USDA Horticultural Crops Production and Genetic Improvement Research Unit populations over both years of the study. Our study was not able to validate or duplicate these results; no significant SSC QTL on chromosome Ra01 were detected within individual years or the combined analysis across years.

A single possible candidate gene (Ra_g7190) coding for a sucrose-binding protein was identified 555 kb away from the closest significant marker at 29,208,288 bp on chromosome Ra02. The sucrose binding protein is strongly associated with cells that actively transport sucrose to young sink leaves and the cotyledon sink cells of soybean [*Glycine max* (L.) Merr.], however, it is involved in a number of different cell types and possibly functions as a monitor of sucrose concentration and uptake in sink tissues (Grimes et al., 1992; Ayre, 2011). However, the QTL on Ra02 for SSC exhibited low LOD scores and was not consistently detected across years, and heritability for SSC was lower than that observed for traits related to acidity. These limitations suggest that rather than pursuing candidate gene validation or developing diagnostic markers for this region, genomic selection (GS) may offer a more effective approach for improving SSC.

### Opportunities for future research

Additional studies should be conducted on the genetic control of sweetness and acidity in fresh-market blackberries to validate the results found in this GWAS and develop diagnostic markers for breeding. Isolating and phenotyping each of the individual sugars and acids would be a useful approach to refine these results and validate or identify additional QTL for sweetness and acidity. Biparental linkage mapping in populations segregating for fruit acidity may refine the fruit acidity locus and reveal whether there is a true deleterious effect of having more than one copy of the low acid allele. Candidate gene sequences of low and high acid genotypes should be compared to identify any nonsynonymous mutations or structural variations that may affect fruit acidity. RNA sequencing of low and high acid genotypes may also reveal differential expression of potential candidate genes within the 2.8 Mb fruit acidity and pH QTL on Ra05.

Data from this research may also be beneficial for GS model development in blackberry. Genomic selection is powerful approach to predict the merit of new breeding selections for low to moderate heritability traits such as SSC, pH, and TA (Bernardo, 2016). The large datasets presented in this study could be used as a foundational training dataset for future GS models. Furthermore, significant SNPs for SSC and pH/TA could be included in genomic selection models as fixed covariates based on their known importance (Rice and Lipka, 2019).

## Conclusion

This is the first study to report estimates of heritability and marker-trait associations for SSC, pH, and TA in *Rubus* using GWAS. Using 1257 replicate samples from 301 fresh-market blackberry genotypes collected over three years, we found broad-sense heritabilities of 61%, 67%, and 70% for SSC, pH, and TA, respectively. We identified four SNPs significantly associated with SSC on chromosome Ra02 and a 3.7 Mb region with 187 SNPs associated with pH and/or TA on chromosome Ra05. The SNP most strongly associated with SSC explained 7.2% of observed phenotypic variance, while the two SNPs most strongly associated with fruit acidity explained 13.6% and 11.5% of observed phenotypic variance for pH and TA, respectively. A sucrose binding protein 555 kb downstream of the most significant SNP on chromosome Ra02 was proposed as a potential candidate gene for SSC. Seven possible candidate genes were identified within 1 Mb of the pH/TA locus with functional annotations related to fruit acidity including including homologs of MYB1, PEPC, malate synthase, and ALMT9. This work provides important insights on the genetic control of sweetness and acidity in tetraploid blackberries. Furthermore, the genotypic and phenotypic datasets reported in this study can be used to develop diagnostic molecular markers for low fruit acidity and to train genomic selection models for enhanced sweetness and reduced acidity in fresh-market blackberries.

## Data Availability

The original contributions presented in the study are publicly available. This data can be found here: Genome Database for Rosaceae repository, accession number tfGDR1069.

## Supplementary material

Supplementary Figure 1 QQ-plots for **(a)** pH, **(b)** titratable acidity (TA), and **(c)** soluble solids content (SSC) showing the observed versus expected distribution of p-values for markers on each *R. argutus* chromosome.

Supplementary Figure 2 QQ-plots for analyisis of soluble solids content (SSC) showing the observed versus expected distribution of p-values for markers on each R. argutus chromosome within each year of the study. **(a)** 2019, **(b)** 2020, **(c)** 2021.

Supplementary Figure 3 QQ-plots for analyisis of pH showing the observed versus expected distribution of p-values for markers on each R. argutus chromosome within each year of the study. **(a)** 2019, **(b)** 2020, **(c)** 2021.

Supplementary Figure 4 QQ-plots for analyisis of titratable acidity (TA) showing the observed versus expected distribution of p-values for markers on each R. argutus chromosome within each year of the study. **(a)** 2019, **(b)** 2020, **(c)** 2021.

Supplementary Figure 5 Manhattan plots showing results from genome-wide association analysis of **(a)** soluble solids content (SSC), **(b)** pH, and **(c)** titratable acidity (TA) within individual years (from 2019-2021) in fresh-market blackberries.

Supplementary Table 1 Number of replicates of each blackberry genotype harvested at the University of Arkansas System Fruit Research Station in Clarksville, AR during 2019, 2020, and 2021. Normalized best linear unbiased estimates (BLUEs) for soluble solids content (SSC) and pH were calculated from data obtained in all three years, while titratable acidity (TA) BLUEs were calculated from samples collected in 2019 and 2020. Predicted genotypes for the two SNPs most strongly associated with pH and TA at 4,448,123 and 4,448,155 bp on chromosome Ra05 are also provided.

Supplementary Table 2 Trait- and model-specific significance thresholds calculated by permutation testing (1,000 iterations) in GWASpoly.

Supplementary Table 3 Significant marker-trait associations for soluble solids content (SSC), pH, and titratable acidity (TA) discovered using genome-wide association analysis using additive and simplex dominance (1-dom-alt and 1-dom-ref) models of gene action.

Supplementary Table 4 Significant marker-trait associations for soluble solids content (SSC), pH, and titratable acidity (TA) within each year discovered using genome-wide association analysis using additive and simplex dominance (1-dom-alt and 1-dom-ref) models of gene action. SSC and pH were measured in three years (2019-2021), while TA was measured only in 2019 and 2020.

## References

[B1] Ayre B. G. (2011). Membrane-transport systems for sucrose in relation to whole-plant carbon partitioning. Mol. Plant 4, 377–394. doi: 10.1093/mp/ssr014, PMID: 21502663

[B2] Bai Y. Dougherty L. Li M. Fazio G. Cheng L. Xu K. (2012). A natural mutation-led truncation in one of the two aluminum-activated malate transporter-like genes at the *Ma* locus is associated with low fruit acidity in apple. Mol. Genet. Genomics 287, 663–678. doi: 10.1007/s00438-012-0707-7, PMID: 22806345

[B3] Bates D. Mächler M. Bolker B. Walker S. (2015). Fitting linear mixed-effects models using *lme4* . J. Stat. Softw 67, 1–48. doi: 10.18637/jss.v067.i01

[B4] Bernardo R. (2016). Bandwagons I, too, have known. Theor. Appl. Genet. 129, 2323–2332. doi: 10.1007/s00122-016-2772-5, PMID: 27681088

[B5] Boudehri K. Bendahmane A. Cardinet G. Troadec C. Moing A. Dirlewanger E. (2009). Phenotypic and fine genetic characterization of the *D* locus controlling fruit acidity in peach. BMC Plant Biol. 9, 59. doi: 10.1186/1471-2229-9-59, PMID: 19445673 PMC2698847

[B6] Brůna T. Aryal R. Dudchenko O. Sargent D. J. Mead D. Buti M.. (2023). A chromosome-length genome assembly and annotation of blackberry (Rubus argutus, cv. “Hillquist. G3 Genes Genomes Genet. 13, jkac289. doi: 10.1093/g3journal/jkac289, PMID: 36331334 PMC9911083

[B7] Cao K. Zheng Z. Wang L. Liu X. Zhu G. Fang W. (2016). Genome-wide association study of 12 agronomic traits in peach. Nat. Commun. 7, 13246. doi: 10.1038/ncomms13246, PMID: 27824331 PMC5105138

[B8] Chizk T. M. Clark J. R. Johns C. Nelson L. Ashrafi H. Aryal R.. (2023). Genome-wide association identifies key loci controlling blackberry postharvest quality. Front. Plant Sci. 14. doi: 10.3389/fpls.2023.1182790, PMID: 37351206 PMC10282842

[B9] Clark J. R. (2005). Intractable traits in eastern U.S. blackberries. HortScience 40, 7. doi: 10.21273/HORTSCI.40.7.1954

[B10] Clark J. R. Finn C. E. (2014). Blackberry cultivation in the world. Rev. Bras. Frutic. 36, 46–57. doi: 10.1590/0100-2945-445/13

[B11] Clark J. R. Stafne E. T. Hall H. K. Finn C. E. (2007). Blackberry breeding and genetics. Plant Breed. Rev. 29, 19–144. doi: 10.1002/9780470168035.ch2

[B12] Danecek P. Auton A. Abecasis G. Albers C. A. Banks E. DePristo M. A.. (2011). The variant call format and VCFtools. Bioinformatics 27, 2156–2158. doi: 10.1093/bioinformatics/btr330, PMID: 21653522 PMC3137218

[B13] De Angeli A. Baetz U. Francisco R. Zhang J. Chaves M. M. Regalado A. (2013). The vacuolar channel VvALMT9 mediates malate and tartrate accumulation in berries of *Vitis vinifera* . Planta 238, 283–291. doi: 10.1007/s00425-013-1888-y, PMID: 23645258

[B14] Dunteman A. N. Threlfall R. T. Clark J. R. Worthington M. L. (2018). Evaluating consumer censory and composition attributes of Arkansas-grown fresh-market blackberries. Discov. Student J. Dale Bump. Coll. Agric. Food Life Sci. 19, 1.

[B15] Earl D. A. vonHoldt B. M. (2012). STRUCTURE HARVESTER: a website and program for visualizing STRUCTURE output and implementing the Evanno method. Conserv. Genet. Resour. 4, 359–361. doi: 10.1007/s12686-011-9548-7

[B16] Etienne A. Génard M. Lobit P. Mbeguié-A-Mbéguié D. Bugaud C. (2013). What controls fleshy fruit acidity? A review of malate and citrate accumulation in fruit cells. J. Exp. Bot. 64, 6. doi: 10.1093/jxb/ert035, PMID: 23408829

[B17] Evanno G. Regnaut S. Goudet J. (2005). Detecting the number of clusters of individuals using the software STRUCTURE: A Simulation Study. Mol. Ecol. 14, 2611–2620. doi: 10.1111/j.1365-294X.2005.02553.x, PMID: 15969739

[B18] Fan-Chiang H. J. Wrolstad R. E. (2010). Sugar and nonvolatile acid composition of blackberries. J. AOAC Int. 93, 3. doi: 10.1093/jaoac/93.3.956, PMID: 20629401

[B19] Finn C. E. Clark J. R. (2017). “Cultivar development and selection,” in Blackberries and their hybrids. Eds. Hall H. K. Funt R. C. (CABI, Boston, MA), 63–92.

[B20] Foster T. M. Bassil N. V. Dossett M. Worthington M. L. Graham J. (2019). Genetic and genomic resources for *Rubus* breeding: a roadmap for the future. Hortic. Res. 6, 116. doi: 10.1038/s41438-019-0199-2, PMID: 31645970 PMC6804857

[B21] Garrison E. Marth G. (2012). Haplotype-based variant detection from short-read sequencing. Genomics 2, 1–9. doi: 10.48550/arXiv.1207.3907

[B22] Gerard D. (2021). Pairwise linkage disequilibrium estimation for polyploids. Mol. Ecol. Resour 21, 1230–1242, 2130–2142. doi: 10.1111/1755-0998.13349, PMID: 33559321

[B23] Gerard D. Ferrão L. F. V. Garcia A. A. F. Stephens M. (2018). Genotyping polyploids from messy sequencing data. Genet 210. doi: 10.1534/genetics.118.301468, PMID: 30185430 PMC6218231

[B24] Grimes H. D. Overvoorde P. J. Ripp K. Franceschi V. R. Hitz W. D. (1992). A 62-kD sucrose binding protein is expressed and localized in tissues actively engaged in sucrose transport. Plant Cell 4, 1561–1574. doi: 10.1105/tpc.4.12.1561, PMID: 1467654 PMC160242

[B25] Guan Y. Peace C. Rudell D. Verma S. Evans K. (2015). QTLs detected for individual sugars and soluble solids content in apple. Mol. Breed. 35, 135. doi: 10.1007/s11032-015-0334-1

[B26] Holland J. B. (2024). Don't BLUP twice. G3: Genes|Genomes|Genetics 14, jkae250. doi: 10.1093/g3journal/jkae250, PMID: 39558791 PMC11631391

[B27] Hu D. G. Li Y. Y. Zhang Q. Y. Li M. Sun C. H. Yu J. Q.. (2017). The R2R3-MYB transcription factor MdMYB73 is involved in malate accumulation and vacuolar acidification in apple. Plant J. 91, 3. doi: 10.1111/tpj.13579, PMID: 28423209

[B28] Hu D. G. Sun C. H. Ma Q. J. You C. X. Cheng L. Hao Y. J. (2016). MdMYB1 regulates anthocyanin and malate accumulation by directly facilitating their transport into vacuoles in apples. Plant Physiol. 170, 3. doi: 10.1104/pp.15.01333, PMID: 26637549 PMC4775115

[B29] Johns C. A. Silva A. Chizk T. M. Nelson L. Clark J. R. Aryal R.. (2025). Genetic control of prickles in tetraploid blackberry. G3 Genes|Genomes|Genetics 15, jkaf065. doi: 10.1093/g3journal/jkaf065, PMID: 40108825 PMC12135004

[B30] Kafkas E. Koşar M. Türemiş N. Başer K. H. C. (2006). Analysis of sugars, organic acids and vitamin C contents of blackberry genotypes from Turkey. Food Chem. 97, 4. doi: 10.1016/j.foodchem.2005.09.023

[B31] Lee W. P. Stromberg M. P. Ward A. Stewart C. Garrison E. P. Marth G. T. (2014). MOSAIK: A hash-based algorithm for accurate next-generation sequencing short-read mapping. PloS One 9, 3. doi: 10.1371/journal.pone.0090581, PMID: 24599324 PMC3944147

[B32] Lerceteau-Köhler E. Moing A. Guérin G. Renaud C. Petit A. Rothan C.. (2012). Genetic dissection of fruit quality traits in the octoploid cultivated strawberry highlights the role of homoeo-QTL in their control. Theor. Appl. Genet. 124, 1059–1077. doi: 10.1007/s00122-011-1769-3, PMID: 22215248 PMC3304055

[B33] Li C. Dougherty L. Coluccio A. E. Meng D. El-Sharkawy I. Borejsza-Wysocka E.. (2020). Apple ALMT9 requires a conserved C-terminal domain for malate transport underlying fruit acidity. Plant Physiol. 182, 2. doi: 10.1104/pp.19.01300, PMID: 31772076 PMC6997694

[B34] Liu J. Zhou M. (2018). The *ALMT* gene family performs multiple functions in plants. Agron 8, 2. doi: 10.3390/agronomy8020020

[B35] Migicovsky Z. (2020). Tasting improvement in fruit flavor using genomics. New Phytol. 226, 1539–1540. doi: 10.1111/nph.16591, PMID: 32343417

[B36] Myers A. L. Threlfall R. T. Howard L. R. Brownmiller C. R. Clark J. R. Worthington M. L.. (2023). Identifying Unique quality attributes of Arkansas-grown fresh-market blackberries. ACS Food Sci. Technol. 3, 816–830. doi: 10.1021/acsfoodscitech.2c00398

[B37] Porebski S. Bailey L. G. Baum B. R. (1997). Modification of a CTAB DNA extraction protocol for plants containing high polysaccharide and polyphenol components. Plant Mol. Biol. Rptr. 15, 8–15. doi: 10.1007/BF02772108

[B38] Pritchard J. K. Stephens M. Donnelly P. (2000). Inference of population structure using multilocus genotype data. Genetics 155, 945–959. doi: 10.1093/genetics/155.2.945, PMID: 10835412 PMC1461096

[B39] Rawandoozi Z. Hartmann T. Byrne D. Carpenedo S. (2021). Heritability, correlation, and genotype by environment interaction of phenological and fruit quality traits in peach. J. Am. Soc Hortic. Sci. 146, 56–67. doi: 10.21273/JASHS04990-20

[B40] Rawandoozi Z. J. Hartmann T. P. Carpenedo S. Gasic K. Da Silva Linge C. Cai L.. (2020). Identification and characterization of QTLs for fruit quality traits in peach through a multi-family approach. BMC Genomics 21, 522. doi: 10.1186/s12864-020-06927-x, PMID: 32727362 PMC7392839

[B41] Rice B. Lipka A. E. (2019). Evaluation of RR–BLUP genomic selection models that incorporate peak genome–wide association study signals in maize and sorghum. Plant Genome 12, 180052. doi: 10.3835/plantgenome2018.07.0052, PMID: 30951091 PMC12962346

[B42] Rosyara U. R. De Jong W. S. Douches D. S. Endelman J. B. (2016). Software for genome-wide association studies in autopolyploids and its application to potato. Plant Genome 9, 2. doi: 10.3835/plantgenome2015.08.0073, PMID: 27898814

[B43] Shaw D. V. (2019). Response to selection and associated changes in genetic variance for soluble solids and titratable acids contents in strawberries. J. Amer. Soc Hortic. Sci. 115, 5. doi: 10.21273/JASHS.115.5.839

[B44] Shin J.-H. Blay S. Graham J. McNeney B. (2006). Ldheatmap : an R function for graphical display of pairwise linkage disequilibria between single nucleotide polymorphisms. J. Stat. Softw 16, 1–9. doi: 10.18637/jss.v016.c03

[B45] Threlfall R. T. Hines O. S. Clark J. R. Howard L. R. Brownmiller C. R. Segantini D. M.. (2016). Physiochemical and sensory attributes of fresh blackberries grown in the southeastern United States. HortScience. 51, 11. doi: 10.21273/HORTSCI10678-16

[B46] United States Department of Agriculture, Economic Research Service. (2024). Fruit and tree nut data. Available online at: https://www.ers.usda.gov/data-products/fruit-and-tree-nuts-data/trade-and-prices-by-category-and-commodity (Accessed January 2025).

[B47] U.S. Food and Drug Administration (2020). Food for human consumption. CFR - code Fed. Regul. title 21. Available online at: https://www.accessdata.fda.gov/scripts/cdrh/cfdocs/cfcfr/cfrsearch.cfm?fr=101.30/ (Accessed June 9, 2021).

[B48] Wang L. Jiang X. Zhao L. Wang F. Liu Y. Zhou H.. (2021). A candidate PpRPH gene of the D locus controlling fruit acidity in peach. Plant Mol. Biol. 105, 321–332. doi: 10.1007/s11103-020-01089-6, PMID: 33128723

[B49] Worthington M. L. (2021). Results of a National Stakeholder Survey of the U.S. Blackberry Industry. Southern Region Small Fruit Consortium – Small Fruit News. Available at: https://smallfruits.org/2021/04/results-of-a-national-stakeholder-survey-of-the-u-s-blackberry-industry/ (Accessed January 2025).

[B50] Zeballos J. L. Abidi W. Giménez R. Monforte A. J. Moreno M. A. Gogorcena Y. (2016). Mapping QTLs associated with fruit quality traits in peach [*Prunus persica* (L.) Batsch] using SNP maps. Tree Genet. Genomes 12, 37.

[B51] Zhen Q. Fang T. Peng Q. Liao L. Zhao L. Owiti A.. (2018). Developing gene-tagged molecular markers for evaluation of genetic association of apple *SWEET* genes with fruit sugar accumulation. Hortic. Res. 5, 14. doi: 10.1038/s41438-018-0024-3, PMID: 29581882 PMC5859117

[B52] Zurn J. D. Driskill M. Jung S. Main D. Yin M. H. Clark M. C.. (2020). A Rosaceae family-level approach to identify loci influencing soluble solids content in blackberry for DNA-Informed breeding. Genes Genomes Genet. 10, 10. doi: 10.1534/g3.120.401449, PMID: 32769135 PMC7534445

