# Supplementary figures and images for: Genetic control of sweetness and acidity in blackberry
Source: Front Plant Sci. 2025 Jul 25;16:1569492. doi: 10.3389/fpls.2025.1569492 (PMC12331689; doi:10.3389/fpls.2025.1569492)

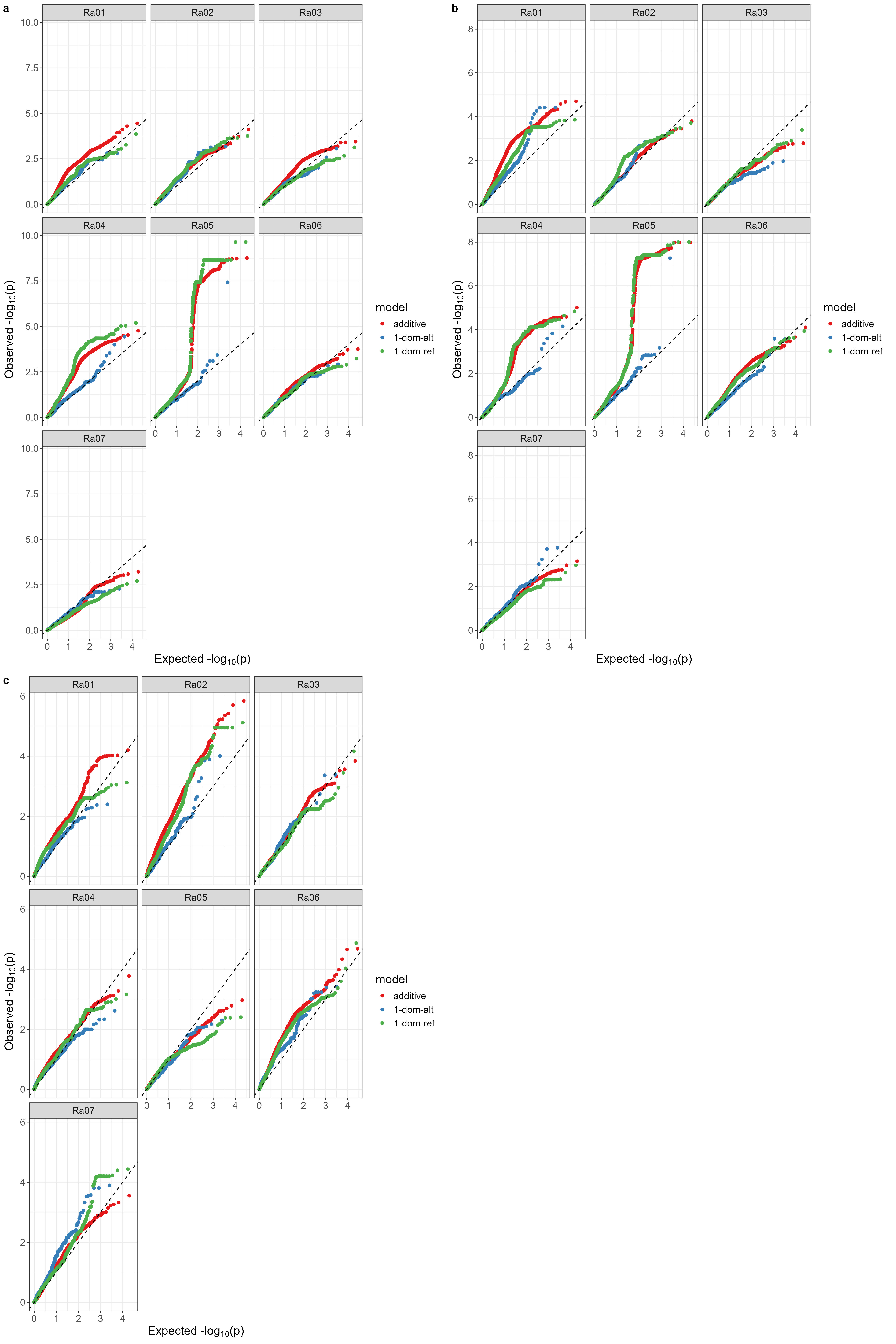

Supplement: Supplementary Figure 1 — QQ-plots for (a) pH, (b) titratable acidity (TA), and (c) soluble solids content (SSC) showing the observed versus expected distribution of p-values for markers on each R. argutus chromosome. [file Image1.jpeg]

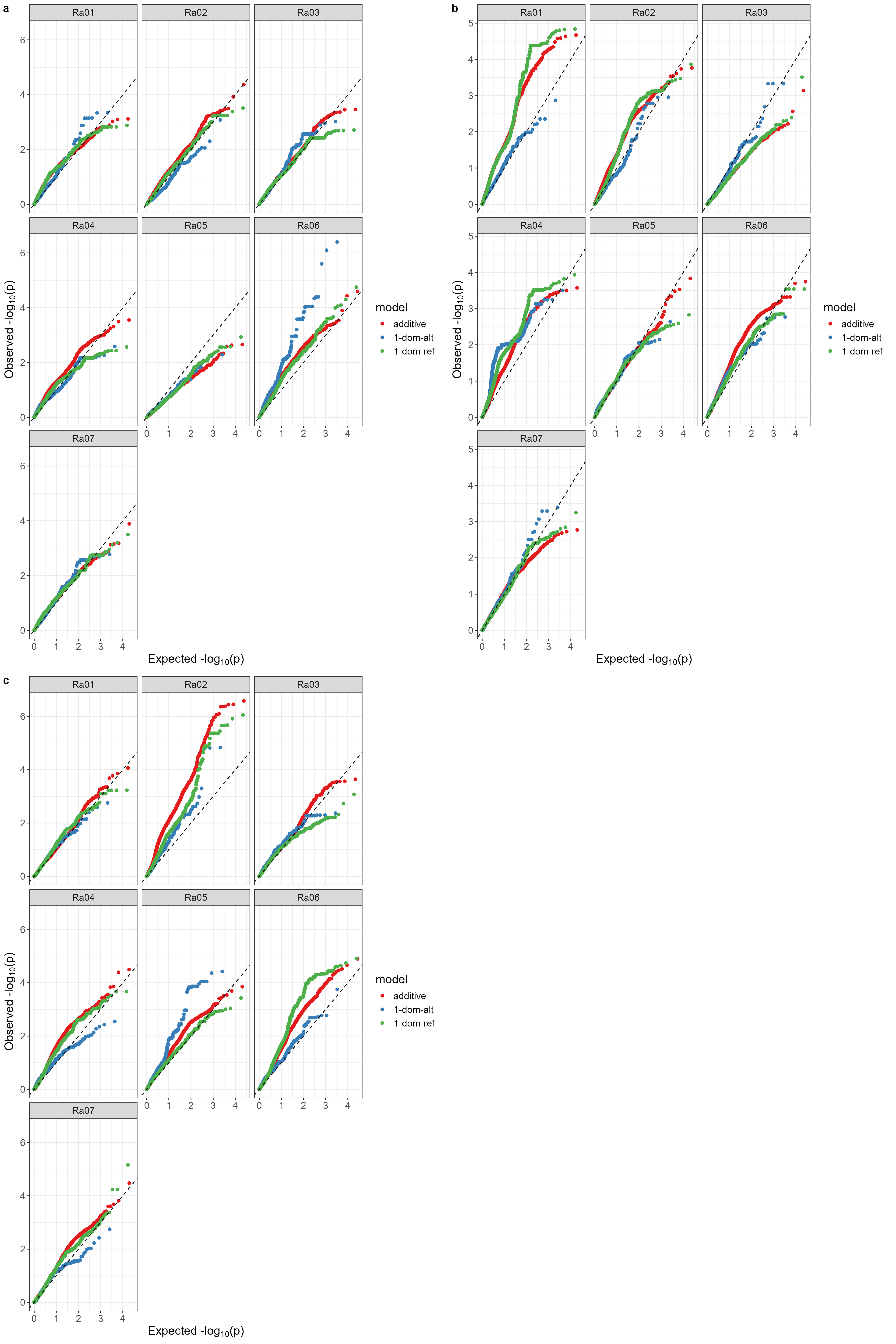

Supplement: Supplementary Figure 2 — QQ-plots for analyisis of soluble solids content (SSC) showing the observed versus expected distribution of p-values for markers on each R. argutus chromosome within each year of the study. (a) 2019, (b) 2020, (c) 2021. [file Image2.jpeg]

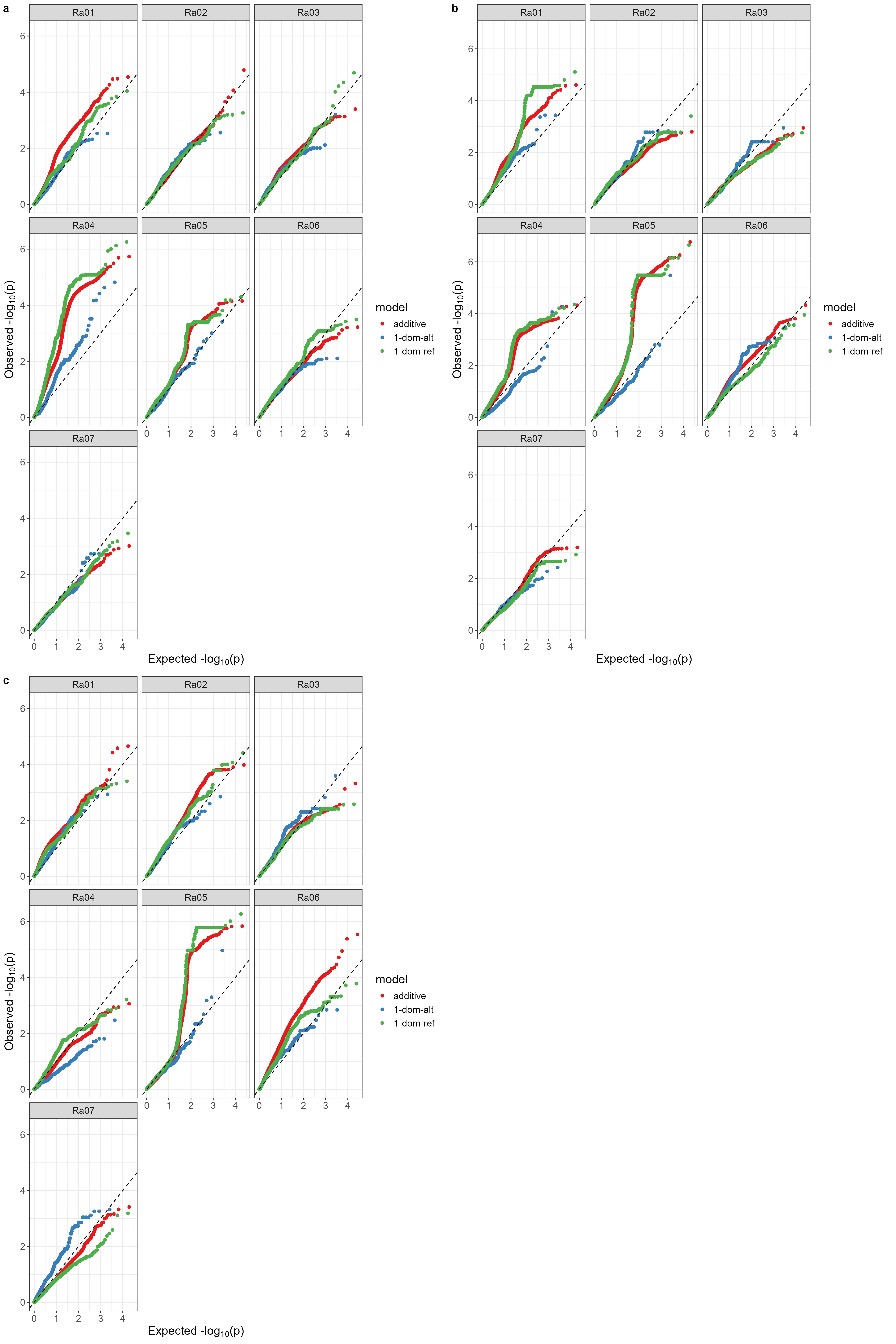

Supplement: Supplementary Figure 3 — QQ-plots for analyisis of pH showing the observed versus expected distribution of p-values for markers on each R. argutus chromosome within each year of the study. (a) 2019, (b) 2020, (c) 2021. [file Image3.jpeg]

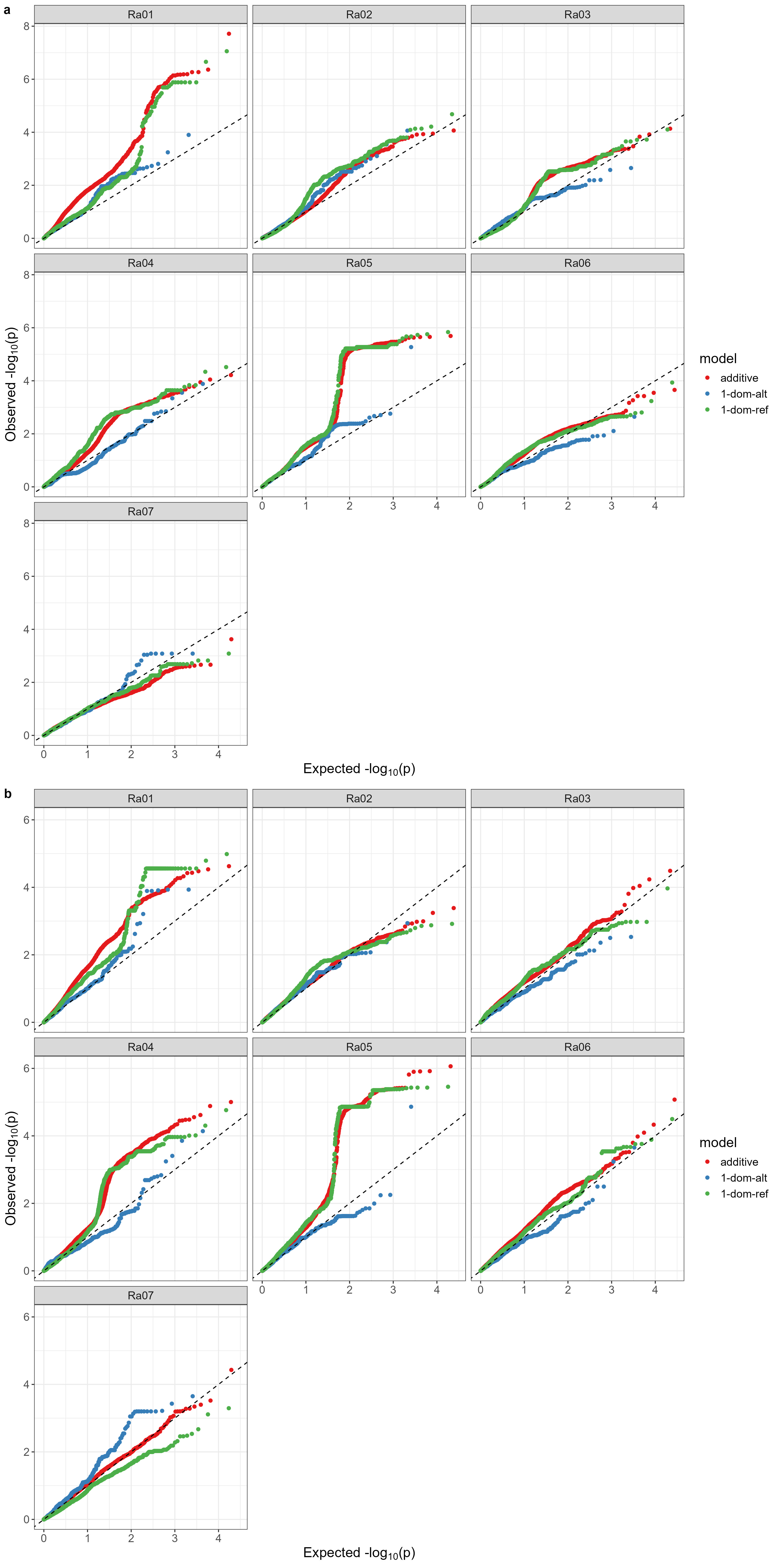

Supplement: Supplementary Figure 4 — QQ-plots for analyisis of titratable acidity (TA) showing the observed versus expected distribution of p-values for markers on each R. argutus chromosome within each year of the study. (a) 2019, (b) 2020, (c) 2021. [file Image4.jpeg]

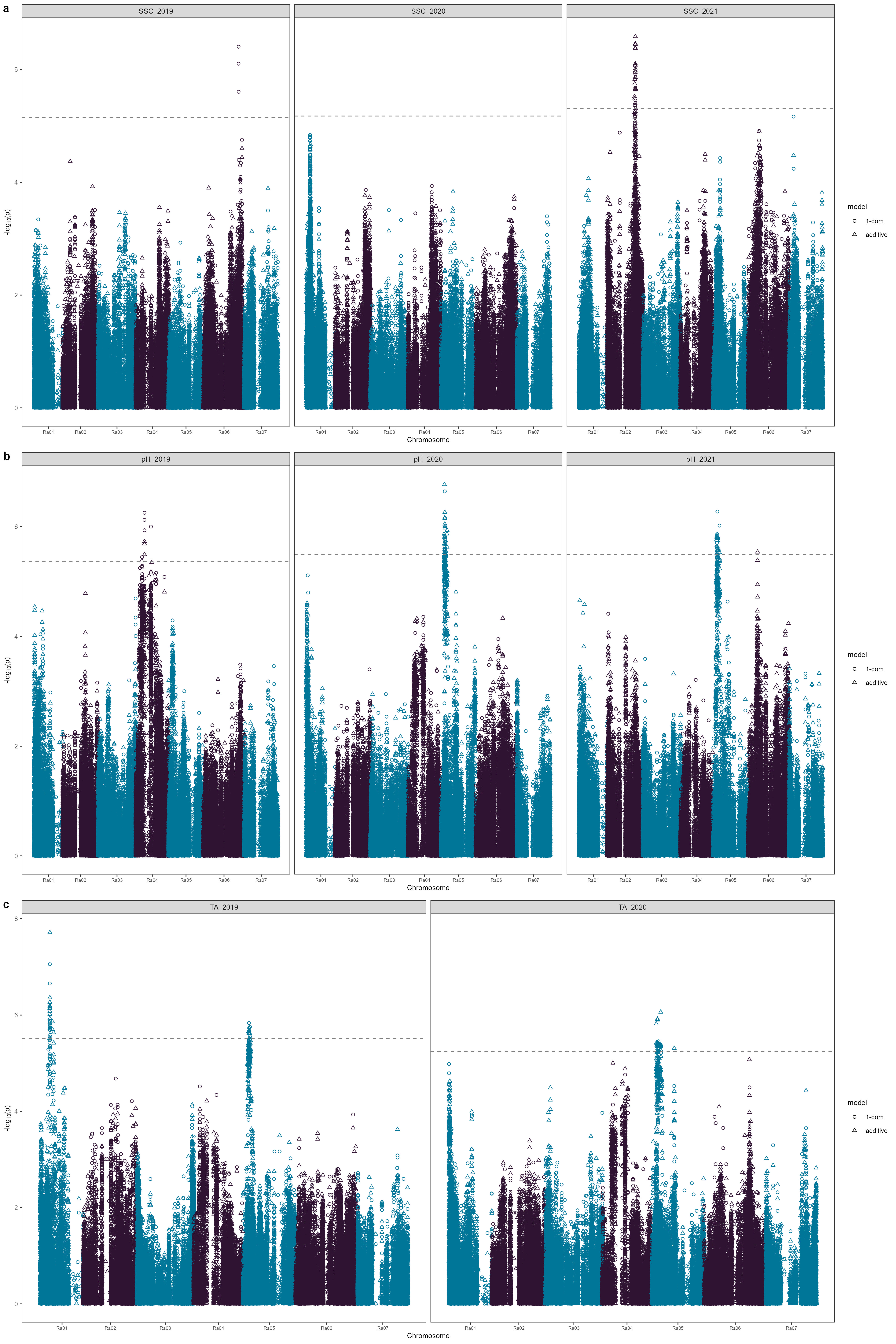

Supplement: Supplementary Figure 5 — Manhattan plots showing results from genome-wide association analysis of (a) soluble solids content (SSC), (b) pH, and (c) titratable acidity (TA) within individual years (from 2019-2021) in fresh-market blackberries. [file Image5.jpeg]
